# Aspergillosis Complicating Severe Coronavirus Disease

**DOI:** 10.3201/eid2701.202896

**Published:** 2021-01

**Authors:** Kieren A. Marr, Andrew Platt, Jeffrey A. Tornheim, Sean X. Zhang, Kausik Datta, Celia Cardozo, Carolina Garcia-Vidal

**Affiliations:** Johns Hopkins School of Medicine, Baltimore, Maryland, USA (K.A. Marr, J.A. Tornheim, S.X. Zhang, K. Datta);; National Institutes of Health, Bethesda, Maryland, USA (A. Platt);; Hospital Clinic of Barcelona, Barcelona, Spain (C. Cardozo, C. Garcia-Vidal)

**Keywords:** aspergillosis, Aspergillus, fungi, complications, severe viral pneumonia, coronavirus disease, COVID-19, severe acute respiratory syndrome coronavirus 2, SARS-CoV-2, respiratory infections, fungal infections, zoonoses

## Abstract

Aspergillosis complicating severe influenza infection has been increasingly detected worldwide. Recently, coronavirus disease–associated pulmonary aspergillosis (CAPA) has been detected through rapid reports, primarily from centers in Europe. We provide a case series of CAPA, adding 20 cases to the literature, with review of pathophysiology, diagnosis, and outcomes. The syndromes of pulmonary aspergillosis complicating severe viral infections are distinct from classic invasive aspergillosis, which is recognized most frequently in persons with neutropenia and in other immunocompromised persons. Combined with severe viral infection, aspergillosis comprises a constellation of airway-invasive and angio-invasive disease and results in risks associated with poor airway fungus clearance and killing, including virus- or inflammation-associated epithelial damage, systemic immunosuppression, and underlying lung disease. Radiologic abnormalities can vary, reflecting different pathologies. Prospective studies reporting poor outcomes in CAPA patients underscore the urgent need for strategies to improve diagnosis, prevention, and therapy.

Invasive aspergillosis is frequently recognized in persons who have severe immunosuppression, especially that associated with hematologic malignancies and transplantation. It is characterized by hyphal invasion through bronchial or lower airway tissues, with potential vascular invasion and hallmark radiographic findings reflective of hemorrhage and necrosis. However, *Aspergillus* species cause a broader constellation of pulmonary disease, pathologically characterized by inflammatory disease in the airway and acute and chronic invasion, largely depending on host risks (*1*). Much recent work has focused on describing epidemiology and significance of aspergillosis occurring after severe viral infections, especially influenza and coronavirus disease (COVID-19).

Aspergillosis associated with severe influenza virus infection (influenza-associated aspergillosis, IAA) was reported in 1951, when Abbott et al. described fatal infection in a woman with cavitary invasive pulmonary aspergillosis noted on autopsy (*2*). Scattered reports appeared in thereafter; Adalja et al. summarized 27 cases in the literature during 1952–2011, which reported predominance after influenza A infection, associated lymphopenia, and occurring in persons of a broad age range (14–89 years), but with little underlying lung disease (*3*). There were increased numbers of cases reported during and after the 2009 influenza A(H1N1) pandemic (*3*–*10*). In 2016, Crum-Cianflone summarized 57 cases from literature; most (70%) were associated with H1N1 influenza (*11*). Invasive aspergillosis was described, with complicating tracheobronchitis noted in 15.8%. Reported cases (68/128) during 1952–2018 were summarized by Vanderbeke et al. (*12*).

An increased understanding of IAA emerged from large cohort studies performed after 2015. One 7-year retrospective study in intensive care units (ICUs) in Belgium and the Netherlands reported rates varying from 14% in immunocompetent persons to 31% in immunocompromised persons (*13*). Within the influenza-infected cohort, male sex, hematologic malignancy, high acute physiological assessment and chronic health evaluation II (APACHE II) score, and corticosteroid use were associated with IAA, whereas underlying diabetes was associated with lower risks. Cohort studies conducted in Canada, China, and Taiwan reported similar risk profiles and that incidence of disease varied according to season and viral epidemiology (*14*–*17*). Despite these data, 2 recent survey studies reported that risk recognition is poor outside of countries in Europe (*18*,*19*). Only 63% of critical care physicians responding to an international survey were familiar with IAA, and differences were notable between physicians in the United States (17%) and Europe (58%) (*19*). Similarly, a US Centers for Disease Control and Prevention–sponsored survey of infectious diseases practitioners reported that only 26% of 114 respondents were familiar with IAA (*18*).

An increased number of reports described a similar syndrome associated with severe COVID-19 (*20**–**45*). In this study, we add to this literature, report 20 additional cases from 2 centers in Spain and the United States and provide a review of literature describing the emerging entity of COVID-19–associated pulmonary aspergillosis (CAPA).

## Methods

### Case Series

Cases of CAPA were identified during March–June 2020 at Johns Hopkins University (Baltimore, MD, USA) and Hospital Clinic of Barcelona (Barcelona, Spain) by review of microbiologic and infectious diseases consult data, with approval of the institutional review boards of both institutions. Cases were defined as recovery of *Aspergillus* species from respiratory fluids (tracheobronchial secretions, sputum, bronchoalveolar lavage [BAL]) or positive (index ≥1) serum or BAL markers, identified with work-up for possible secondary pneumonia, typically clinically indicated with new fever or respiratory decompensation with new focal infiltrates on chest radiograph or computed tomography (CT) scan. Results for a Fungitell β-d Glucan Assay (https://www.fungitell.com) were reported when available but did not suffice to establish case diagnosis; 60 pg/mL was considered intermediate and ≥80 pg/mL was considered positive. Neither center used PCR-based testing for fungal infections during this period. Charts were reviewed to summarize demographic, clinical, and outcomes data, including day of hospitalization and ICU admission relative to reported onset of symptoms. World Health Organization (WHO) ordinal scale scores (0–8) at diagnosis of CAPA were estimated (*46*).

### Analyses

We calculated descriptive statistics for all data. These values are shown as frequencies, means (±SD), medians (with ranges), and proportions.

## Results

### Characteristics of Cases

Patient-level data were compiled in cases recognized before June 2020 at Johns Hopkins Medical Center and Hospital Clinic of Barcelona (Table). Demographics mirrored those described for poor overall outcomes; advanced age and underlying diseases of hypertension and pulmonary disease predominated. Two patients had an underlying immunosuppressive disease. The most common immunosuppressing agents associated with CAPA included systemic or inhaled steroids, most frequently for adjunctive management of COVID-19 related inflammatory disease. CAPA was recognized a median of 11 days after symptom onset and 9 days after ICU admission. Most of these patients were hospitalized during stages characterized by inflammation or acute respiratory distress syndrome or afterwards, with lung injury, in the ICU and required respiratory support. Thus, WHO ordinal classifications at CAPA diagnoses were ≥5 (*46*).

**Table:** Case series of aspergillosis complicating severe viral pneumonia in 20 persons with coronavirus disease*

Patient no.	Age, y/sex	Underlying condition	Previous IS	Days from ICU to CAPA	Days from symptoms to CAPA	CT result†	BDG assay	GM assay	GM BAL	Respiratory culture	Outcome
1	71/F	COPD, ESRD	Neg	8	9	GGO, nodules^A^	Pos	Neg	ND	*A. fumigatus*‡	Survived
2	56/F	COPD, DM, HTN, CHF, obesity	Neg	2	2	GGO, nodules^B^	ND	Pos	ND	ND	Survived
3	70/F	HTN	Neg	10	10	ND	Pos	Pos	ND	ND	Died
4	68/F	HTN, dementia	Neg	2	4	ND	ND	Neg	ND	*A. fumigatus*‡	Survived
5	69/M	HTN	Neg	12	13	GGO, consolidations^C^	ND	Neg	ND	*A. niger*‡	Died
6	55/M	Quadriplegia, HTN, CAD	Neg	−1	1	GGO, consolidations^D^	Neg	Neg	ND	*A. fumigatus*‡	Survived
7	85/F	DM, HTN, ESRD	Neg	9	11	Consolidations, nodules, cavities^E^	Pos	Pos	ND	*A. fumigatus*‡	Survived
8	50/F	ESRD, asthma	Tocilizumab	23	ND	ND	Pos	Neg	ND	*A. fumigatus*‡	Survived
9	45/F	MS, SLE, asthma	Ocrelizumab	12	23	GGO, consolidations, airway wall thickening^F^	Int	Neg	ND	*A. fumigatus*§	Survived
10	57/F	DM	Neg	4	13	ND	Neg	Neg	ND	*A. fumigatus*‡	Survived
11	57/M	Lymphoma	Rituximab	22	5	ND	Int	Neg	ND	*A. fumigatus*‡	Survived
12	61/M	HTN	Neg	16	11	ND	Neg	Pos	ND	Neg	Died
13	75/F	HTN, COPD, DM, obesity	Neg	12	14	ND	ND	ND	ND	*A. fumigatus*‡	Survived
14	56/F	Asthma	Tocilizumab	2	8	ND	ND	Neg	ND	*A. fumigatus*‡	Survived
15	70/M	HTN	Neg	3	9	Consolidations^G^	ND	ND	ND	*A. fumigatus*‡, *A. niger*‡	Survived
16	76/M	COPD	Steroids	11	14	ND	ND	Neg	ND	*A. fumigatus*†, *A. niger*†	Survived
17	73/M	HTN, DM	Steroids	7	13	GGO, consolidations^H^	ND	Neg	ND	*Aspergillus* spp‡	Survived
18	78/M	HTN, prosthetic mitral valve	Steroids, siltuximab	14	16	ND	ND	ND	ND	*Aspergillus* spp.‡	Survived
19	56/F	None	Tocilizumab, anakinra	9	19	ND	ND	ND	ND	*A. terreus*§	Survived
20	81/M	DM, HTN, cirrhosis	Steroid	24	39	Consolidations, cavity^I^	ND	Neg	Pos	*A. niger*§	Survived
Total	55% F, mean age 65.5 y (range 45–81 y)	HTN 60%; COPD/asthma 35%; DM 30%, ESRD 15%	Steroids (4/20, 20%); other (6/20, 30%)	Median 9.5 (range −1 to 24)	Median 11 (range 1–42)		pos 4/9 (44%); neg 3/9 (33%); int 2/9 (22%)	pos 4/16 (25%); neg 12/16 (75%)	0/1	Culture pos 3 BAL, 14 airways	Died 3/20 (15%)

**Figure 1:**
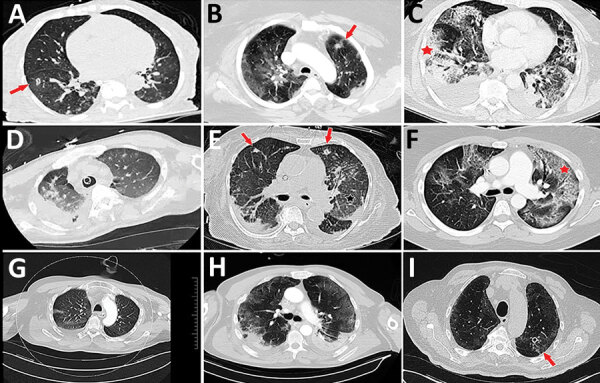
Representative computed tomography (CT) scans for 9 patients with aspergillosis complicating severe viral pneumonia in patients with coronavirus disease. Scans were obtained at or around diagnosis of coronavirus disease–associated pulmonary aspergillosis in this series of patients, described in the Table (https://wwwnc.cdc.gov/EID/article/27/1/20-2896-T1.htm). Corresponding case-patients are indicated with lettered superscripts in the radiology column of Table 1. Examples of nodules and cavitating nodules are indicated by red arrows, and prominent airway thickening and bronchiectasis in ground glass opacities are indicated by red stars.

*Patients 1–12 were from the United States, and patients 13–20 were from Spain. BAL, bronchioalveolar lavage; BGD, β-d glucan assay; CAD, coronary artery disease; CAPA, coronavirus disease–associated pulmonary aspergillosis; CHF, congestive heart failure; COPD, chronic obstructive pulmonary disease; CT, computed tomography; DM, diabetes mellitus; ESRD, end-stage renal disease; GM, galactomannan; GGO, ground glass opacities; HTN, hypertension; Int, intermediate; IS, immunosuppression; MS, multiple sclerosis; ND, not done; neg, negative; pos, positive; SLE, systemic lupus erythematosus.  †Results are from CT scan unless indicated otherwise. Chest radiograph images representing descriptions are shown in Figure 1. Corresponding panels are indicated as superscript letters. ‡Tracheal aspirate/sputum. §BAL.

In cases for which CT scans were performed, radiographic reports generally described a mixture of findings attributable to the virus (ground glass opacities and crazy-paving), findings consistent with airway inflammation and mucous plugging (bronchiectasis, airway wall thickening and irregularity, bronchiectasis), and radiographic findings consistent with airway-invasive disease (consolidations, tree-in-bud nodules) (Table; Figure 1). In some cases, larger nodules with necrosis and cavitation were noted. Although nodular necrosis with cavitation was described, no radiographic reports highlighted findings that are classically associated with angioinvasive disease (ground glass attenuation described as halos) (*47*).

Bronchoscopy was rare, and diagnosis was most frequently supported by tracheal aspirate culture; few patients had positive serum biomarkers. Seventeen (85%) cases were identified by positive culture; most (12/17, 71%) were identified by detection of *A. fumigatus*. Although rarely used, results of Fungitell β-d glucan assays were more frequently positive compared with serum galactomannan assays. All but 2 patients were given intravenous antifungal drugs, which included voriconazole, posaconazole, or liposomal amphotericin B. One patient (#2) was not treated for findings of a nodule and positive serum galactomannan result, was extubated, and survived. Another patient (#12) had diagnosis of CAPA established 1 day before death and never received antifungal therapy.

### Synopsis of Literature

**Figure 2:**
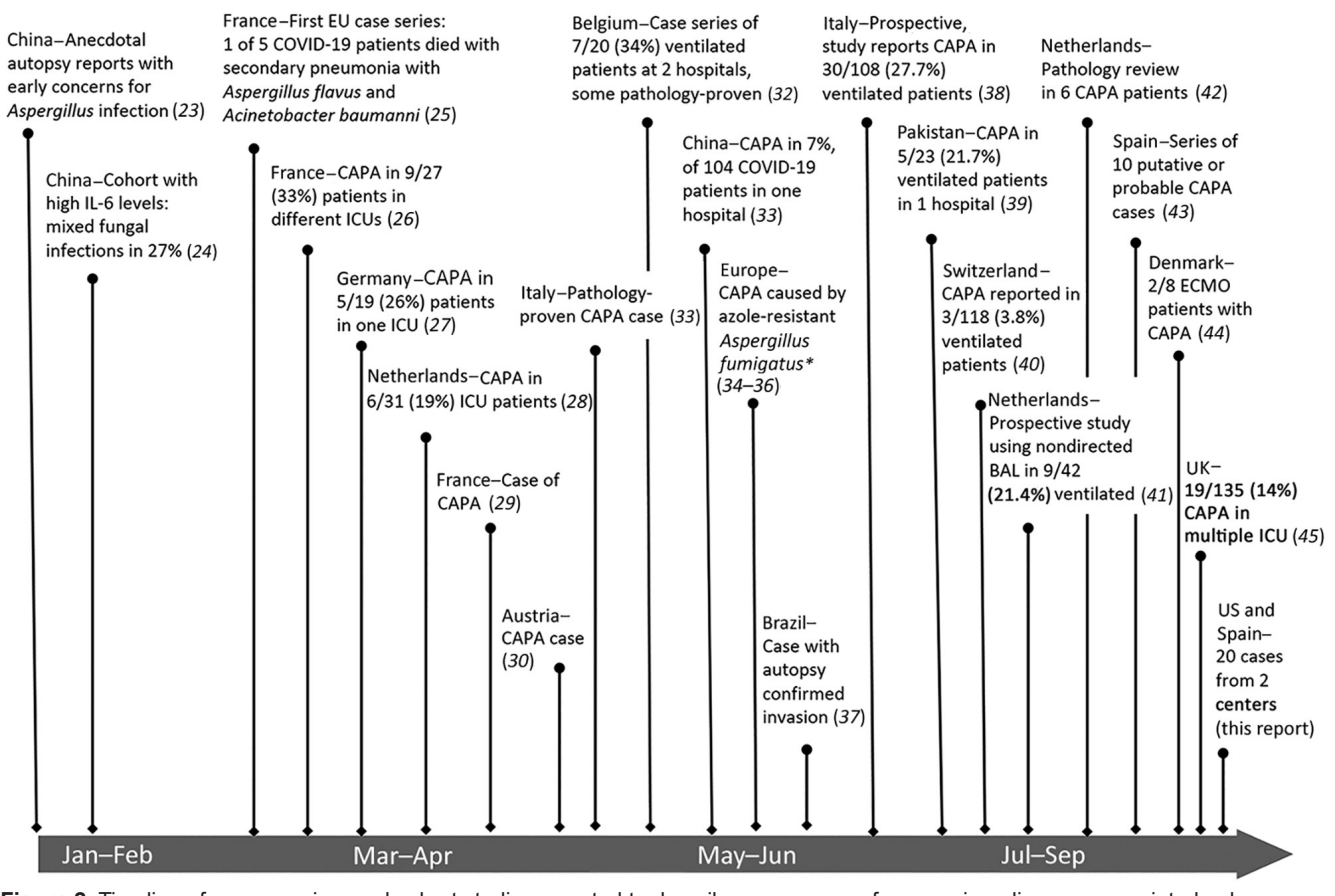
Timeline of cases, series, and cohort studies reported to describe emergence of coronavirus disease–associated pulmonary aspergillosis. Reports from China are indicated according to relative times that patients were given care; case series describing CAPA cases are depicted according to approximate time publication became available (preprint or publication), except as indicated (*). BAL, bronchioalveolar lavage; CAPA, coronavirus disease–associated pulmonary aspergillosis; ECMO, extracorporeal membrane oxygenation; EU, European Union; ICU, intensive care unit; IL-6, interleukin 6.

Evidence of secondary aspergillosis developing in persons infected with severe acute respiratory syndrome coronavirus 2 was first evident in China but emphasized more clearly by case series from Europe. We provide a timeline of studies describing secondary aspergillosis occurring in persons with COVID-19, an entity that has become referred to CAPA (Figure 2) (*23**–**45*). Early reports from China noted frequent CT findings suggestive of invasive aspergillosis but provided few microbiologic or clinical details. Although without specific citation, a US Department of Defense document on COVID-19 noted that there were anecdotal communications of invasive aspergillosis documented in postmortem examinations in China (*23*). Use of empirical antifungal drugs was frequent; in a large study evaluating risk factors for death, »50% of persons had secondary infections, and antifungal therapy was administered in 22% (*21*,*22*). Radiographic descriptions were suggestive of invasive disease; in a study describing radiography in 51 patients, 11 (22%) had nodules with halos or reverse halos (*20*). Without manifestations of patient-specific data, these cohort studies nonetheless indicated that there were substantial issues with secondary fungal infections in persons who had WHO stage II–III disease. In a study from China that evaluated outcomes of persons who had increased levels of serum interleukin 6, mixed fungal infections occurred in 27.1% of 48 critically ill patients (*24*). In another study from China, *Aspergillus* species were recovered from respiratory fluids in 14% of COVID-19 patients (*24*).

Patient-level descriptions emerged quickly in small case series from Europe. In the first case series from Europe for COVID-19, a total of 1 of 5 patients had alveolar infiltrates on chest radiograph and *Aspergillus* spp. cultured from tracheal aspirate (*25*). Thereafter, rapid reports from centers in Austria, Belgium, France, Germany, the Netherlands, and Italy emerged (Figure 2). Differences in diagnostic methods and case definitions generated a wide degree of variability, and incidences ranged from 3.8% to 34% of persons admitted to ICUs (*24*–*45*). One center in China reported rates based on the denominator of persons hospitalized because of COVID-19 and that CAPA developed in 7% of 104 patients who had CAPA (*33*). Bronchoscopy was variably and infrequently performed, but frequent positive results for lavage culture, galactomannan, and *Aspergillus* PCR were observed, and there was visual presence of thick mucoid secretions, sometimes with evidence of bronchial inflammation, such as ulcers (*33*). Using multivariable analysis, Wang et al. reported that advanced age, chronic lung disease, previous positive results for the β-d glucan assay, antimicrobial drug exposure, and mechanical ventilation to be risks for CAPA in persons who had COVID-19 (*33*). Histopathologic evidence of fungal invasion has been noted in some, but not all, patients who met CAPA definitions by airway culture or lavage galactomannan and underwent lung biopsy or autopsy (*32*,*38*,*42*). CAPA caused by an azole-resistant *A. fumigatus* isolate was first described in the Netherlands and then in the United Kingdom and France (*34**–**36*).

Results of 3 studies that used a prospective design provided the most accurate estimates of incidence, timing, and clinical usefulness. A prospective, multicenter study that used screening with serum biomarkers and bronchoscopy for 108 mechanically ventilated patients in Italy reported that 30 (27.7%) persons met CAPA criteria (median of 4 days after ICU admission and 14 days after COVID-19 diagnosis) (*38*). Higher mortality rates were noted for CAPA patients than for controls; there were trends to improved survival and decreased follow-up galactomannan levels after antifungal therapy. Another study from the Netherlands applied nondirected BAL by using a closed-circuit suction catheter at a median of 2 days (range 0–8 days) after mechanical ventilation and reported 9/42 (21.4%) patients met criteria for CAPA on the basis of culture or galactomannan BAL positivity; patients with CAPA had longer duration of ICU admission (*41*). Finally, another prospective study that used enhanced screening with blood and respiratory samples, antigen assays (galactomannan enzyme immunoassay [GM EIA] and β-d glucan assay), and an *Aspergillus* PCR reported that 19/135 patients met diagnostic criteria for CAPA when concurrent radiographic abnormalities were considered (*45*).

## Discussion

Despite decades of case reporting and large cohort studies, many clinicians still fail to recognize that *Aspergillus* species can cause destructive inflammatory and invasive pathology in persons who have severe influenza, mistakenly ascribing culture results to benign airway colonization (*18*,*19*). With this in mind, the rapid recognition of CAPA, as described by reports from multiple centers (Figure 2), probably reflects previous learning and heightened awareness in centers in Europe and the clinical diligence that arises when encountering a new and unknown entity. We add 20 cases to the accumulating literature describing CAPA. Multiple pathophysiologic, clinical, and diagnostic considerations have emerged from observations reported to date.

**Figure 3:**
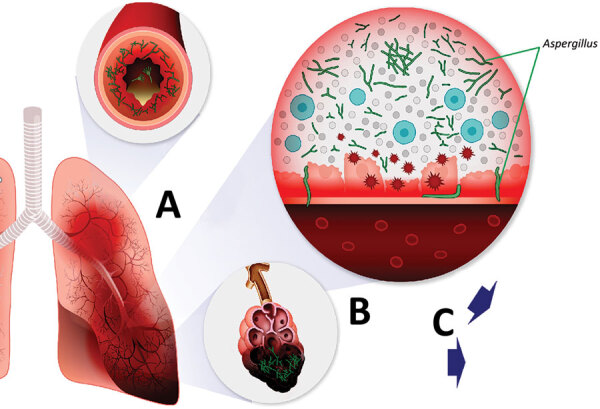
Schematic of coronavirus disease–associated pulmonary aspergillosis. *Aspergillus* conidia in airway are cleared poorly because of ≥1 defects in primary pulmonary immunity or secondary defenses, enabling conidial germination into hyphal morphotypes, which elicit increased inflammatory responses in the airway and potential invasion into the lungs. A mixed constellation of inflammatory to invasive airway disease, and invasive pulmonary aspergillosis leads to multiple manifestations, including tracheobronchitis and obstructive pneumonia, and complications of invasive fungal pneumonia (nodules, necrosis with cavities, pleural invasion). A) Airways. *Aspergillus* overgrowth causes pathologic airway inflammation and excess mucous production. B) Alveoli. Hyphal growth causes invasive pneumonia. C) Invasive aspergillosis tracheobronchitis postobstructive bacterial pneumonia.

First, pathophysiology of disease is distinct in this context, and not necessarily similar to invasive aspergillosis that occurs in classically immunosuppressed persons. Although it is broadly understood that *Aspergillus* species cause allergic manifestations, such as allergic bronchopulmonary aspergillosis, and a severe invasive pneumonia with potential angioinvasion, forms of chronic necrotizing or semiinvasive *Aspergillus* pulmonary disease are less well understood. These types of infections occur in persons who have more chronic immunosuppression, especially that related to prolonged steroids and chronic obstructive pulmonary disease. Common pathophysiology of these syndromes involves poor clearance of conidia, enabling bronchial inflammation and invasion, manifesting with distinct radiographic and clinical findings characteristic of airway invasion with slower development of necrosis, and exuberant and chronic tracheobronchitis, frequently with lack of angioinvasion, which limits performance of serum-based diagnostics (*48*). Mounting evidence suggests that severe respiratory virus infections, especially influenza and infection with severe acute respiratory syndrome coronavirus 2, can be complicated by *Aspergillus* airway overgrowth with pulmonary infection similarly characterized by mixed airway inflammation and bronchial invasion (Figure 3).

Distinguishing between benign airway colonization and potential disease caused by *Aspergillus* spp. has always been difficult because conidia are common inhabitants of airways and do not always cause inflammatory or invasive disease. To improve the process of obtaining information about what constitutes disease, early efforts have been directed toward standardizing diagnostics and definitions by using a similar approach, as for IAA, which eliminates the classic immunocompromising host criteria and relies on BAL and serum antigen results to define certainty of disease (*49**–**51*). Cases reported in this study would be considered probable CAPA if tracheal aspirate or sputum cultures met microbiologic criteria. Although more efforts are required to clarify definitions, clinicians should understand that definitions are not meant to provide clinical guidance, but to support a metric to compare epidemiology and clinical trial data. Three prospective cohort studies suggest that treatment with antifungal drugs might improve clinical outcomes (*38*,*41*,*45*), but definitive evidence of clinical usefulness necessitates larger comparative studies that use antifungal drugs for prevention or early therapy.

Most published studies suggest that CAPA occurs in »20%–30% of the most severely ill, mechanically ventilated COVID-19 patients (*24*–*45*). Perhaps the most accurate estimates of incidence emerged from 3 studies that deployed enhanced prospective screening and provided incidence estimates of 14%–20% and poor outcomes that might potentially be improved by use of antifungal therapy (*38*,*41*,*45*). Another study reported a particularly low rate of CAPA (3.8%) (*40*). Diagnostic differences probably contribute to variability in estimates.

Performance of diagnostic testing is variable depending on immunopathogenesis of disease. Persons who have extensive invasion into and beyond airways show positive serum GM EIA results more frequently than persons who had disease restricted to the endobronchial lumen. For this reason, sensitivity of the serum GM EIA assay is highest in hematology/oncology patients, ranging from 60% to 80%, but lower in ICU patients, estimated to be 30%–50% (*48*–*52*). In CAPA cases, serum GM EIAs have been infrequently positive. In our case series, results of β-d glucan assays were more frequently positive, but false-positive results would be anticipated because of to cross-reactivity (*52*). A prospective study reported a relatively large proportion of cases with positive results for β-d glucan assays for patients who had candidiasis (*38*). When applied to BAL, results for GM EIA appear positive more frequently, and some investigators reported potential utility of quantitative PCR or GM lateral flow tests in lavage (*24**–**45*). However, because none of these tests were developed and optimized for the nonhematology context, performance could be variable with different cutoff values.

Despite diagnostic limitations, several studies point to utility in routine use of fungal biomarkers and early screening in persons who have COVID-19, especially directed toward BAL. Lei et al. examined residual serum samples by using a β-d glucan assay and GM in 181 COVID-19 patients who had oxygen saturation <94% or respiratory rates >29 breaths/min, and reported positive results in the β-d glucan assay for 32 (17.7%) of 181 patients and positive results in the GM EIA for 14 (7.7%) of 181 patients sampled (*53*). In that study, most positive results occurred after 14 days of COVID-19 symptoms, which is consistent with the timing of recognized CAPA. Although their retrospective study was limited by lack of clinical and outcomes data, findings suggest that at least some cases might be identified by more aggressive use of a biomarker screening strategy. This suggestion was shown more definitively in prospective studies that evaluated BAL or lavage from a close-circuit system, with antifungal therapy applied for lavage GM EIA positivity potentially leading to improved outcomes (*38**,**41**,**45*). Deploying such a strategy might be limited in some centers by complexity of assays and difficulties in obtaining bronchoscopy because of viral infectivity.

Radiographic manifestations might be best understood when one considers that CAPA can be a constellation of mixed airway and invasive diseases. In our series and other reports, radiographic appearance varied from early evidence of airway inflammation and invasion (irregular airways to centrilobular nodules) to airway necrosis; this necrosis was most frequently characterized by cavitary nodules and progressive consolidation (*33*). Corresponding histopathology can be varied, including diffuse alveolar damage, with or without clear fungal invasion (*32**,**43*).

Although many questions linger, emerging evidence supports the conclusion that *Aspergillus* species cause mixed pathology in COVID-19 patients, ranging from airway inflammation to semiacute or acute bronchial invasion, similar, in most part, to that observed with severe influenza infections. Increased efforts are needed to determine the best ways to prevent, diagnose, and treat *Aspergillus* disease associated with COVID-19.
